# Diagnostic Performance and Additional Value of Elastosonography in Focal Breast Lesions: Statistical Correlation between Size-Dependant Strain Index Measurements, Multimodality-BI-RADS Score, and Histopathology in a Clinical Routine Setting

**DOI:** 10.1155/2014/396368

**Published:** 2014-03-09

**Authors:** Lukas Ebner, Harald M. Bonel, Adrian Huber, Steffen Ross, Andreas Christe

**Affiliations:** ^1^Department for Diagnostic, Interventional and Pediatric Radiology, University Hospital Inselspital Bern, Freiburgstraße 10, 3010 Bern, Switzerland; ^2^Institute of Forensic Medicine, University of Zürich, Virtopsy, 8057 Zurich, Switzerland

## Abstract

*Objective.* To evaluate the diagnostic benefit of real-time elastography (RTE) in clinical routine. Strain indices (SI) for benign and malignant tumors were assessed. *Methods.* 100 patients with 110 focal breast lesions were retrieved. Patients had mammography (MG), ultrasound (US), and, if necessary, MRI. RTE was conducted after ultrasound. Lesions were assessed with BI-RADS for mammography and ultrasound. Diagnosis was established with histology or follow-up. *Results.* SI for BI-RADS 2 was 1.71 ± 0.86. Higher SI (2.21 ± 1.96) was observed for BI-RADS 3 lesions. SI of BI-RADS 4 and 5 lesions were significantly higher (16.92 ± 20.89) and (19.54 ± 10.41). 31 malignant tumors exhibited an average SI of 16.13 ± 14.67; SI of benign lesions was 5.29 ± 11.87 (*P* value <0.0001). ROC analysis threshold was >3.8 for malignant disease. Sensitivity of sonography was 90.3% (specificity 78.5%). RTE showed a sensitivity of 87.1% (specificity 79.7%). Accuracy of all modalities combined was 96.8%. In BI-RADS 3 lesions RTE was able to detect all malignant lesions (sensitivity 100%, specificity 92.9%, and accuracy 93.9%). *Conclusions.* RTE increased sensitivity and specificity for breast cancer detection when used in combination with ultrasound.

## 1. Introduction

Breast cancer is one of the most common cancers occurring in women [1], but sensitive diagnostic imaging modalities that detect cancer early are frequently limited by their low specificity. In addition to digital mammography, ultrasound has been established as a valuable tool for making early diagnoses, especially for focal masses in dense breast glands [2]. The first clinical uses of real-time strain elastography for mammary lesions were described between 1997 and 2003 [3–5]. Real-time elastography (RTE) can be rapidly and easily performed along with a B-scan and Doppler ultrasound during the same session. This procedure can aid in identifying the lesion's morphological features and in obtaining information regarding the tissue's mechanical characteristics [6]. Itoh et al. [7] established a scoring system to morphologically classify lesions in a manner analogous to the Breast Imaging and Reporting Data System (BI-RADS) [8].

RTE can be used to monitor mechanical tissue properties via an ultrasound probe that calculates the strain produced by an externally applied force. Using the combined autocorrelation method (CAM), data regarding tissue displacement prior to and following the compression is measured and merged with the conventional B-mode image [7, 9]. In addition to the morphological information, the result of these two measurements is the so-called “strain index,” representing a semiquantitative evaluation obtained by comparing the strain levels of different normal-appearing areas of the breast with the strain level of the lesion on the elasticity maps. A higher strain index (SI) indicates less strain in the measured lesions. This score also correlates with the stiffness of a palpable mass in physical breast examinations. Importantly, most malignant tumors are less compressible than the surrounding tissue, similar to what can be observed with manual palpations.

Although a conventional ultrasound can differentiate focal lesions from normal tissue to some extent, histopathology is the standard of reference. A conventional sonography in combination with RTE should therefore increase the sensitivity for breast cancer diagnoses [10]. One aim was therefore to further consolidate results obtained in those previous studies.

We examined the impact of RTE to a morphologic ultrasound examination along with a mammogram on sensitivity and specificity for malignant disease. Furthermore, the influence of the diameter of the lesion on the strain index was examined. Finally, we correlated histopathologic tumor entity and the strain indices.

## 2. Materials and Methods

**Figure 1:**
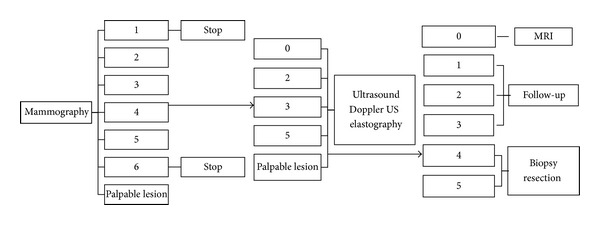
Principal study design: imaging work-up of lesions and classification by mammography, ultrasound, and real-time elastography. MRI was conducted if necessary.

This study was approved by the ethics committee and was performed according to the standards of good clinical practice (GCP). Standards for the reporting of diagnostic accuracy studies (STARD criteria) were applied. Because additional RTE was performed along with the routine ultrasound, written informed consent from the patients was waived. From the women undergoing routine mammography screening between February 2008 and February 2009 a total of 105 consecutive patients were retrospectively recruited. The principle study design is displayed in Figure 1. Inclusion criteria were a BI-RADS classification based on mammography of 0, 2, 3, 4, and 5, with an additional workup with ultrasonography. All of the patients were female and had a median age of 53 years (range: 26 to 87 years). All of the imaging examinations were classified in consensus by two radiologists with 10 and 2 years of experience in breast imaging according to the BI-RADS classification referring to mammography and sonography (Table 1). BI-RADS classification was conducted in every single case for mammography. Patients with known, focal malignancies (BI-RADS 6) and patients with normal negative mammography (BI-RADS 1) were not included. Five patients were excluded due to missing compliance for follow-up examination. All other BI-RADS scores were further investigated by sonography and Doppler ultrasound and were then reclassified (Figure 1). 26 patients with lesions still needing additional imaging evaluation (BI-RADS 0 and uncertain BI-RADS 3 or 4) underwent further breast MR-imaging and were reclassified again (Figure 1). All patients with a final BI-RADS score of 4 or 5 received a diagnostic 16 G core biopsy or open surgery was performed to determine the histopathologic diagnosis, as recommended by the American College of Radiology [8]. For BI-RADS 3 lesions a 6-month follow-up mammography and ultrasound was performed. If there was no change in the follow-up exam, another mammography and ultrasound were conducted after another 6 months. A stable lesion was reexamined after one year to prove the benign nature. Changes in lesion characteristics resulted in upgrading of the BI-RADS assessment and appropriate further actions were taken (histology). The remaining BI-RADS 2 lesions were examined in a regular follow-up period of 2 years.

## 3. Mammography

Each patient was questioned regarding their familial risk factors, and a clinical exam (inspection, palpation) preceded the imaging procedures. The findings were documented on a standardized evaluation form. In patients under 40 years of age, mammography was indicated if there were familial risks or clinical findings. Mammography was available for all of the patients in two standard projections, craniocaudal and oblique. The same physicians who performed the sonography and real-time elastosonography also immediately viewed all mammography results with the patient in the waiting room. The density of the breast was classified according to the American College of Radiology (ACR) type, and the lesions were classified according to the BI-RADS system.

## 4. Ultrasound 

An ultrasound was performed following the mammography on the same day. An ultrasound exam was indicated for the following patient or case characteristics: dense breasts of ACR Type III and IV, palpable lesions (BI-RADS 0), or a suspicious lesion detected on mammography (BI-RADS 2–5).

A diagnostic ultrasound system made by Hitachi Medical Corporation was used for all examinations (Hitachi model Hi-Vision 900; Hitachi Medical Corporation, Tokyo, Japan). Linear probes were used for both ultrasound breast imaging and elastography. These probes provide a steady compression and a homogenous strain in the area of interest. The present study was performed using the Hitachi probe model EUP L-74 M (13 MHz). The patients were positioned on a standard patient stretcher and slightly elevated at 20 degrees. To improve the sonographic conditions, the patients were asked to position their hands behind their neck. Following the application of a conventional ultrasound gel, the different sectors of the breast were examined for focal lesions using the linear probe. The breast glands were searched systematically for focal lesions using a radial scanning pattern. Each breast was divided into 5 sections: 4 outer quadrants of the same size and the retroareolar region. According to the ACR-BI-RADS ultrasound classification criteria lesions were considered malignant if one of the following criteria was evident: irregular shape, spiculated margins, irregular lesion boundary, and echo pattern (i.e., complex echo patterns like both echoic and anechoic components, complicated, cystic patterns). Posterior acoustic behavior, architectural distortion of the surrounding tissue, calcification, and vascularity were also taken into consideration. In addition, the focal lesions were classified into groups according to their diameter, which was determined using B-mode sonography. The classifications were stratified into ten-millimeter groups as follows: 1–10 mm, 11 to 20 mm, and >21 mm.

**Figure 2:**
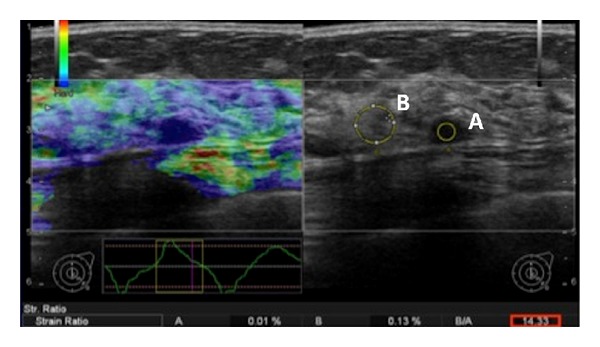
The right side of the figure shows an ultrasonographic image (B-mode) of a 56-year-old female patient suffering from an invasive ductal carcinoma. The first region of interest (ROI A) is positioned in the center of the hypoechoic tumor and was compared with the reference ROI B, which contains the normal glandular tissue. On the left side of the image, the stiffness of the different tissues is color-coded and superimposed on the grayscale image. The carcinoma is less compressible than the surrounding normal tissue. The strain index in this case was 14.3, suggesting malignancy.

**Figure 3:**
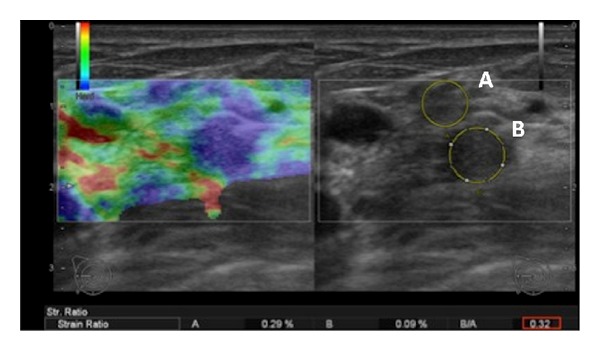
An example of fibrocystic changes in a 35-year-old female. The B-mode ultrasound image on the right indicates a hypoechoic nodular lesion. On the color-coded elastography, the lesion appears to be less strained than the surrounding tissues. The region of interest (ROI A) is positioned in normal parenchyma, and the second ROI B is positioned within the lesion. The strain index is 0.32; therefore, the lesion is most likely benign.

Elastography was performed after the ultrasound study was conducted. Three strain indices of the suspected lesion were documented in the patient reports. The elastography software by Hitachi indicated the pressure amplitude on the screen to avoid placing too much pressure on the tissue and to get reproducible values. For the elasticity measurements, a region of interest (ROI) was positioned over both, (1) the focal lesion and (2) homogenous breast tissue. The first ROI over the suspected lesion was drawn as large as possible to cover the whole lesion. The latter ROI (at least 2 cm) served as a reference for the software to calculate the tissue strain in the lesion (Figures 2 and 3).

## 5. Magnetic Resonance Imaging

In 26 cases, additional magnetic resonance imaging (MRI) was performed using a 1.5 tesla MRI unit (Siemens Sonata, Erlangen, Germany). The standardized protocol consisted of axial T2-weighted and turbo-inversion-recovery sequences and a 3D T1-weighted gradient echo sequence (fast low-angle shot). The contrast dynamics were determined using an intravenous paramagnetic contrast agent in standard dosage (Gadolinium; Dotarem 0.5 mmol/mL; Guerbet, Roissy, France). Six measurements including precontrast imaging lasting about 1 minute each were acquired continuously. Diffusion-weighted imaging and transverse fat-suppressed T1-weighted turbo-spin echo sequences were used to complete the protocol. The matrix size was 320 × 320 for the dynamic imaging and 512 × 512 voxels for the T2-weighted and turbo inversion recovery sequences. Criteria for malignancy were irregular margins, invasion in adjacent structures, initial rise of the kinetic curve, and washout. Further, every structural distortion was considered suspicious.

## 6. Statistical Analysis

Lesions classified as BI-RADS 4 and 5 were considered malignant and BI-RADS 3 lesions were assessed as benign entity for sensitivity, specificity, and accuracy calculations. To investigate the performance of real-time elastography in comparison with an established testing method (conventional ultrasound and mammography), the sensitivities and specificities of both modalities for identifying malignant lesions were determined. To compare the performance of real-time elastography (RTE) with conventional ultrasound, the sensitivity and specificity of both methods were calculated for malignant lesions using a two-dimensional contingency table. These results were verified using positive and negative likelihood ratio testing. The comparison between the size-dependent sensitivity, specificity, and accuracy of the conventional ultrasound combined with mammography against real-time elastography was analyzed using the McNemar test for matched, nominal data [11]. Moreover, the different means of the different BI-RADS categories acquired by conventional methods were compared against the measured strain indices using one-way analysis of variance (ANOVA [12]). Given normal distribution of the data, Wilcoxon rank-sum test [12] was performed to compare the strain indices of the malignant and benign lesions and to assess whether their population mean ranks differ. A cutoff strain index for malignant lesions with maximum sensitivity was determined using a receiver operating characteristic (ROC) curve [12]. *P* values less than 0.05 were considered statistically significant. Microsoft Excel 2003 (Microsoft, Redmond, USA) and MedCalc (v7 6.0.0 Ostend, Belgium) were used for statistical evaluations. For a sample size of 31 cancers an increase in sensitivity for malignancy from 90.3% (28 of 31 cancers) to 100% (31 of 31 cancers) could be possible at a significance level of 5% (alpha-error) with a power of 80% (beta-error). In other words, the sample size is large enough to detect 3 additional cancers adding elastography to mammography/sonography with a power of 80%.

## 7. Results

A total of 110 lesions were identified in 100 patients, consisting of 31 malignant (*n* = 28.2%) and 79 benign lesions (71.8%) (Table 2). Among the malignancies, there were 23 ductal carcinomas (74.2%), four lobular carcinomas (12.9%), two mucinous carcinomas (6.5%), one papillary carcinoma (3.2%), and one adenocarcinoma (3.2%). The average lesion size was 14.7 mm (range 4 to 110 mm).

Forty-nine out of 110 lesions measured between 1 and 10 mm (45%). Forty-three lesions measured 10 mm to 20 mm (39%). Eighteen lesions had a diameter greater than 20 mm (16%).

### 7.1. Strain Indices in relation to BI-RADS Classification

Table 3 lists the different BI-RADS scores and the distribution of the mean of the associated strain indices including standard deviation. The mean strain index for BI-RADS 2 lesions was 1.71 ± 0.86 (±standard deviation). A slightly higher score was observed for BI-RADS 3 lesions (2.21 ± 1.96). The strain index of the BI-RADS 4 and 5 lesions demonstrated significantly higher values (16.92 ± 20.89 and 19.54 ± 10.41, resp.). Analysis of variance tested the SI (=strain index) for the different BI-RADS levels together as significant (*P* value <0.0001).

### 7.2. Strain Indices and Histological Diagnosis

**Figure 4:**
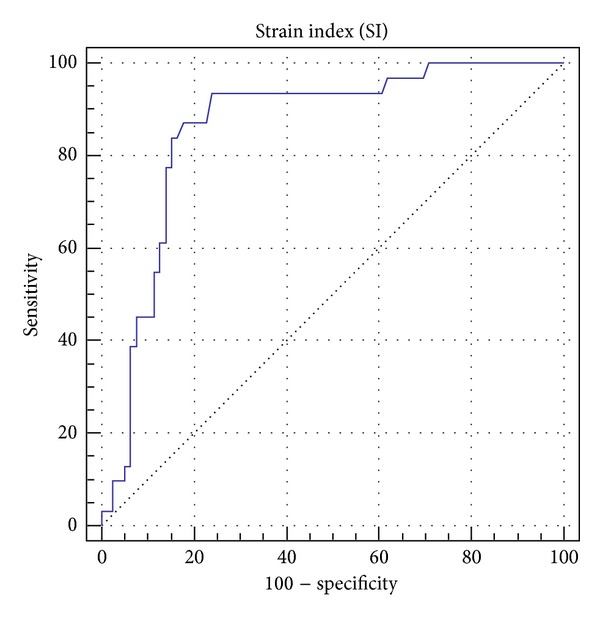
Receiver operating characteristic (ROC): area under the ROC curve = 0.861, standard error = 0.045, 95% Confidence interval = 0.782 to 0.919, and disease prevalence = 28.2%. The ROC analysis shows a cutoff level for malignant disease at an elastography coefficient of >3.8. With this criterion accuracy for malignant disease is highest with a sensitivity of 93.5 (95% C.I. = 78.5–99.0) and specificity of 75.9 (95% C.I. = 65.0–84.9).

**Figure 5:**
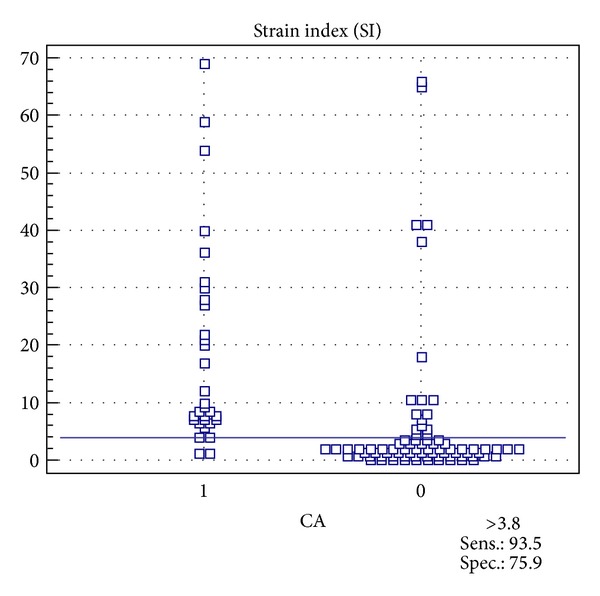
Scatter plot for multivariate data providing the distribution of strain indices associated with malignancy (1) and with benign lesions (0). The cutoff is set at 3.8 as calculated in the ROC analysis.

The 31 malignant tumors featured a mean strain index of 16.13 ± 14.67, whereas the benign tumors (*n* = 79, including BI-RADS 2, 3 and biopsy-proven benign lumps) only showed a mean strain index of 5.29 ± 11.87 (*P* value ≤0.0001). The ductal and lobular carcinomas exhibited the highest scores (17.43 and 45.13, resp.). Mastitis and scars both evinced low scores (2.00 and 1.85 ± 0.92). Examples of benign and malignant disease scores are given in Figures 2 and 3. The ROC analysis indicated a cutoff level for malignant disease at a strain index of 3.8 (Figures 4 and 5). The accuracy of identifying a malignant disease is highest with this criterion, with a sensitivity of 93.5% (95% CI = 78.5%–99.0%) and a specificity of 75.9% (95% CI = 65.0%–84.9%).

### 7.3. Accuracy of Real-Time Elastography and Mammography in Combination with Ultrasound

Pooled sensitivity of mammography and ultrasound for malignancy was 90.3%, which was 3.2% higher than the sensitivity of elastosonography alone (87.1%). The specificity of mammography and conventional ultrasound (78.5%) was 1.4% lower than the specificity of real-time elastography (79.7%). The overall accuracy of mammography and ultrasound for malignancies (81.8%) is the same as for elastosonography alone (Tables 4 and 5). All those findings did not prove to be statistically significant. Therefore elastosonography alone is as accurate as mammography and ultrasound together within our selected study collective.

### 7.4. Size-Dependant Sensitivity and Specificity

Sensitivity drops from 100% for lesions >2 cm to 77.8% for lesions <1 cm for mammography in combination with the ultrasound (US). Similar results arose for RTE alone, except lesions >2 cm showed slightly lower sensitivity of 88.9% with RTE.

### 7.5. Performance of Elastosonography When Combined with Ultrasound and Mammography

McNemar's test did not demonstrate a significant difference in sensitivity, specificity, or accuracy for any size group between the combination of mammography and ultrasound versus elastosonography. But the analysis of variance (ANOVA) showed a highly significant result for the association of BI-RADS categories and the related strain indices (<0.0001). If a conventional method and/or real-time elastography was positive for malignancy, the combined classification counted as positive test result. Using this approach, the detection rate for malignant lesions increased from 28 to 30 (out of 31). In fact, sensitivity advanced from 90.3% to 96.8%. Nevertheless the *P* value remained not significant (0.48). The 30 lesions classified as BI-RADS 3 after mammography and US are displayed in a separate Table (Table 6). RTE solely was able to reclassify the two false negative BI-RADS 3 lesions as malignant (sensitivity 100%). Altogether, the positive and negative likelihood ratios for mammography and ultrasound combined, RTE alone, and the combination of both methods are listed in Tables 4 to 7. A likelihood ratio >1 indicates a positive relation between the test result and disease. A likelihood ration <1 is associated with the absence of disease. Positive and negative likelihood ratios for US, RTE, and the combined testing lie far from 1, indicating practical significance as the posttest probability is little different from the pretest probability.

## 8. Discussion

Ultrasound examinations are important clinical procedures for determining the diagnosis of a breast lesion. This technique is especially important for patients with dense mammary gland tissue (ACR values of 3 and 4) given that the sensitivity of the mammography is low for these patients [13]. Additionally, real-time elastography (RTE) can provide real-time information on tissue composition during the same session as the ultrasound. The strain index is an objective, measurable numerical value, and previous studies have demonstrated a correlation between the BI-RADS categories and strain indices [14]. A mean strain index of 1.71 and 2.21 was calculated for BI-RADS categories 2 and 3, respectively. Those low strain indices for benign lumps correspond with values reported in the literature [3, 15]. Highly suspicious lesions (BI-RADS categories 4 and 5) exhibited strain indices with average values of 16.9 and 19.5, respectively, indicating reduced elasticity. In BI-RADS 3 cases RTE was able to identify all true positive, malignant lesions as good as mammography and ultrasound combined (sensitivity 100%). Given the nature of the BI-RADS-classification, sample size of malignant lesions initially classified as BI-RADS 3 was low (*n* = 2). But the fact that RTE could detect 2 malignant lesions classified as probably benign (BI-RADS 3) with conventional sonography and mammography is promising and is worth further investigation.

The ROC analysis we performed indicated that a strain ratio of 3.8 is the cutoff level for malignant disease in the present study. The mean cutoff value for malignancy that we report is slightly lower than the one observed by Itoh et al. (4.2 ± 0.9) [7]. The study conducted by Cho et al. [16] reported a strain ratio of 3.9 as the cutoff for malignancy. Balleyguier et al. also showed a cutoff point between 3 and 4 [17]. It is worth mentioning that the SR is depending on the manufacturer of the elastography unit [18].

Itoh et al. published a cutoff level for malignancy of 4.2 and used a handmade stabilizer to minimize rotation of the probe on the skin surface maybe influencing the results. Despite the fact that the sample size of Itoh et al. was similar to our study population (111 patients versus 100 patients in our group), Itoh et al. included 52 cases of breast cancer versus 31 cases in our evaluation. Nevertheless, lesion size and histological distribution were quite similar.

The comparison of elastography with conventional ultrasound and mammography, which was performed to determine whether any information was gained by performing real-time elastography, was of particular interest. Conventional ultrasound and mammography exhibited a sensitivity of 90.3% and a specificity of 78.5% for malignancy. In contrast, real-time elastography alone demonstrated a sensitivity of 87.1% and a specificity of 79.7%. The real-time elastography results for mammary lesions as compared with conventional ultrasound findings were similar to previously reported findings. According to Thomas et al., [15] B-mode ultrasound achieved a sensitivity of 91.8% and a specificity of 78%. By combining ultrasound with real-time elastography, a specificity of 91.5% may be achieved. The study by Itoh et al. indicated a sensitivity of 86.5% and a specificity of 89.8% for elastography compared with a lower sensitivity (71.2%) and a higher specificity (96.6%) for ultrasound. As suggested by Itoh et al., we created ten-millimeter categories of lesion size to investigate the connection between lesion size and the accuracy of real-time elastography versus conventional ultrasound. The results are displayed in Tables 4 and 5. For larger masses (≥20 mm), the specificity, and therefore the accuracy, was higher for conventional ultrasound analysis. This result is likely due to the fact that larger lesions have a certain degree of stiffness. By placing the ROI within the periphery of a lesion, away from the probe, the mass in the ROI is less compressible. The mass between the ROI and the probe may neutralize the applied pressure. Therefore, we suggest that the ROI for larger tumors should be placed as close to the probe as possible; however, further studies should be performed to verify this hypothesis. The present test results indicate that higher accuracy for malignancy may be achieved by combining conventional methods with real-time elastography, but a larger study population is needed to get statistically significant results.

Furthermore, an elevated strain index may point towards a special malignant entity. The average strain indices measured for ductal carcinomas were 17.43 ± 13.80 and an average score of 45.13 ± 33.06 was measured for lobular carcinomas. The histopathologic correlation is limited by the prominent variation from the average SI represented by a broad deviation of values. According to Zhi et al., [19] mucinous carcinomas can exhibit benign-appearing strain indices in early elastography examinations. This finding could not be confirmed by our test results or by other evaluations [18]. We found a mean strain index of 7 ± 4.24 in two cases of mucinous carcinomas.

## 9. Limitations

Despite the promising results presented here with respect to the diagnostic power of real-time elastography, some limitations of our study should be considered. The number of histological malignant tumors among the patient sample was relatively small (31 cases, 28.18%), but the prospective evaluation of more patients is ongoing. One limitation in the study is the high number of nonpathologically proved presumed benign lesions (BI-RADS 2 and 3); they compromise 71% from the study cases. Due to this small number of cases in our cohort, it is not possible to provide representative information for all carcinoma entities. Furthermore, it is known that the acquisition and the interpretation of elastography images by radiologists are clearly subjective [20, 21]. Also the measured strain indices showed a broad variation. A recent study demonstrated that breast thickness is also a limiting factor in diagnostic specificity [22].

## 10. Conclusion

Despite the limitations mentioned above, our study demonstrated that real-time elastography is a promising method for increasing the accuracy of conventional sonography in a clinical setting. We were able to show that especially in BI-RADS 3 lesions the use of RTE could increase the sensitivity for malignancy in this category. In a clinical routine setting, we were able to elevate the detection rate of malignant lesions from 28 to 30 (*n* total = 31) suggesting the beneficial implementation of real-time elastography in clinical examinations. In fact, three BI-RADS 3 lesions could be reclassified with RTE and were proven to be malignant. This suggests that the use of RTE is especially valuable for the characterisation of BI-RADS 3 rated lesions.

## Figures and Tables

**Table 1:** ACR BIRADS categories in ultrasound: ultrasound assessment categories as proposed by the ACR (BI-RADS).

0	Need for additional imaging evaluation.
	*The final assessment category cannot be determined by ultrasound. Further imaging studies involve mammography and MRI. *

1	Negative.
	*In this category, no lesions are found on ultrasound. There are no mass forming processes or calcifications present. *

2	Benign findings.
	*Lesions in this category do not require further imaging*.

3	Probably benign finding—short-interval follow-up is recommended.
	*In the ACR recommendations, this category consists of lesions with circumscribed margins, oval shape, and horizontal orientation. Further, complicated cysts may also be placed in this group. *

4	Suspicious abnormality—biopsy should be considered.
	*The ACR states that lesions belonging to this category exhibit a probability of cancer ranging from 3% to 94%. Those lesions are always biopsied at our institution. *

5	Highly suggestive of malignancy—appropriate action should be taken.
	*The risk of cancer is above 95%. Those lesions require core biopsy and/or excision. Staging for lymph node involvement is mandatory. *

6	Biopsy-proven malignancy—appropriate action should be taken.

Copyright Notice

American College of Radiology (ACR) Breast Imaging Reporting and Data System Atlas (BI-RADS Atlas). Reston, VA: ^©^American College of Radiology. 2003. All rights reserved.

(1) The italicized text is explanatory but not part of the BI-RADS assessments and should not be represented as such.

**Table 2:** Elasticity score for individual pathology and comparison between malignant and benign disease.

Histology	*n*=	Mean SI^1^	SD^2^	Median SI
Ductal carcinoma	23	17.43	(±13.80)	9.40
Lobular carcinoma	4	45.13	(±33.06)	59.00
Mucinous carcinoma	2	7.00	(±4.24)	7.00
Adenocarcinoma	1	1.60		1.60
Papillary carcinoma	1	6.47		6.47
Malignant	**31**	**18.69**	**(±17.56)**	**9.40**
Fibrocystic mastopathy	7	15.27	(±18.56)	2.40
Adenosis	7	14.35	(±22.86)	6.10
Fibroadenoma	5	15.57	(±27.70)	4.57
Scar	2	1.85	(±0.92)	1.85
Fatty necrosis	1	10.80		10.80
Mastitis	1	2.00		2.00
Normal	56	2.82	(±5.25)	1.74
Benign	**79**	**5.82**	**(±12.39)**	**2.20**

^1^SI: strain index.

^2^SD: standard deviation.

**Table 3:** BIRADS scores and associated strain indices. Further, the strain indices of malignant and benign lesions are displayed.

	*n*=	Mean SI^1^	SD^2^
Histology			
Malignancy	31	16.31	14.67
Benign lesions	79	5.29	11.87
Total	**110**		
BI-RADS			
2	35	1.71	0.86
3	30	2.21	1.96
4	33	16.92	20.89
5	12	19.54	10.41
Total	**110**		

^1^SI: strain index.

^2^SD: standard deviation.

**Table 4:** Accuracy calculations for mammography and ultrasound in combination.

	TP^1^	FN^2^	TN^3^	FP^4^	Sens.^5^	Spec.^6^	Acc.^7^
>20 mm	9	0	5	4	1.000	0.556	0.778
10–20 mm	12	1	21	9	0.923	0.700	0.767
1–10 mm	7	2	36	4	0.778	0.900	0.878
Total	**28**	**3**	**62**	**17**	**0.903**	**0.785**	**0.818**

^1^TP: true positive; ^2^FN: false negative; ^3^TN: true negative; ^4^FP: false positive; ^5^Sens.: sensitivity; ^6^Spec.: specificity; ^7^Acc.: accuracy.

**Table 5:** Accuracy calculations for real-time sonoelastography scores.

	TP^1^	FN^2^	TN^3^	FP^4^	Sens.^5^	Spec.^6^	Acc.^7^
>20 mm	8	1	4	5	0.889	0.444	0.667
10–20 mm	12	1	22	8	0.923	0.733	0.791
1–10 mm	7	2	37	3	0.778	0.925	0.898
Total	**27**	**4**	**63**	**16**	**0.871**	**0.797**	**0.818**

^1^TP: true positive; ^2^FN: false negative; ^3^TN: true negative; ^4^FP: false positive; ^5^Sens.: sensitivity; ^6^Spec.: specificity; ^7^Acc.: accuracy.

**Table 6:** Accuracy calculations for BI-RADS 3 lesions.

		TP^1^	FN^2^	TN^3^	FP^4^	Sens.^5^	Spec.^6^	Acc.^7^
	*n* = 30	2	0	26	2	1	0.929	0.933

PPV^8^	50.00%	CI 95%	8.30–91.70%	PLR^10^	14	CI 95%^12^	3.68–53.23	
NPV^9^	100.00%	CI 95%	86.65–100.00%	NLR^11^	0			

^1^TP: true positive; ^2^FN: false negative; ^3^TN: true negative; ^4^FP: false positive; ^5^Sens.: sensitivity; ^6^Spec.: specificity; ^7^Acc.: accuracy; ^8^PPV: positive predictive value; ^9^NPV: negative predictive value; ^10^PLR: positive likelihood ratio; ^11^NLR: negative likelihood ratio; ^12^CI: confidence interval.

**Table 7:** Accuracy calculations for the combination of mammography, sonography and RTE.

		TP	FN	TN	FP	Sens.	Spec.	Acc.
	*n*=	30	1	60	19	0.968	0.759	0.818

PPV	61.22%	CI 95%	46.24–74.80%	PLR	4.02	CI: 2.71–5.99		
NPV	98.36%	CI 95%	91.17–99.73%	NLR	0.04	CI: 0.01–0.27		
